# RANKL increases the level of Mcl-1 in osteoclasts and reduces bisphosphonate-induced osteoclast apoptosis *in vitro*

**DOI:** 10.1186/ar2681

**Published:** 2009-04-30

**Authors:** Karen A Sutherland, Helena L Rogers, Denise Tosh, Michael J Rogers

**Affiliations:** 1 Bone & Musculoskeletal Research Programme, School of Medicine & Dentistry, Institute of Medical Sciences, University of Aberdeen, Foresterhill, Aberdeen, AB25 2ZD, UK

## Abstract

**Introduction:**

Bisphosphonates are the most widely used class of drug for inhibiting osteoclast-mediated bone loss, but their effectiveness at preventing joint destruction in rheumatoid arthritis has generally been disappointing. We examined whether the ability of bisphosphonates to induce osteoclast apoptosis and inhibit bone resorption *in vitro *is influenced by the cytokine receptor activator of nuclear factor-kappa B ligand (RANKL), an important mediator of inflammation-induced bone loss.

**Methods:**

Rabbit osteoclasts were treated with the bisphosphonates clodronate or alendronate for up to 48 hours in the absence or presence of RANKL. Changes in cell morphology and induction of apoptosis were examined by scanning electron microscopy, whilst resorptive activity was determined by measuring the area of resorption cavities. Changes in the level of anti-apoptotic proteins, including Mcl-1, Bcl-2, and Bcl-x_>L_, were determined in rabbit osteoclasts and in cytokine-starved mouse osteoclasts by Western blotting.

**Results:**

RANKL significantly attenuated the ability of both clodronate and alendronate to induce osteoclast apoptosis and inhibit bone resorption. Treatment of rabbit osteoclasts with RANKL was associated with an increase in the anti-apoptotic protein Mcl-1 but not Bcl-2. A role for Mcl-1 in osteoclast survival was suggested using osteoclasts generated from mouse bone marrow macrophages in the presence of RANKL + macrophage colony-stimulating factor (M-CSF) since cytokine deprivation of mouse osteoclasts caused a rapid loss of Mcl-1 (but not Bcl-2 or Bcl-x_L_), which preceded the biochemical and morphological changes associated with apoptosis. Loss of Mcl-1 from mouse osteoclasts could be prevented by factors known to promote osteoclast survival (RANKL, M-CSF, tumour necrosis factor-alpha [TNF-α], or lipopolysaccharide [LPS]).

**Conclusions:**

RANKL protects osteoclasts from the apoptosis-inducing and anti-resorptive effects of bisphosphonates *in vitro*. The ability of RANKL (and other pro-inflammatory factors such as TNF-α and LPS) to increase the level of Mcl-1 in osteoclasts may explain the lack of effectiveness of some bisphosphonates in preventing inflammation-induced bone loss.

## Introduction

The molecular mechanisms by which bisphosphonate (BP) drugs inhibit osteoclast-mediated bone resorption have been clarified in recent years [1]. After targeting bone mineral and internalisation by osteoclasts, simple BPs such as clodronate are metabolised intracellularly by osteoclasts to form non-hydrolysable analogues of ATP which induce osteoclast apoptosis [2]. By contrast, the nitrogen-containing BPs such as alendronate and zoledronate do not appear to be metabolised but are potent inhibitors of farnesyl diphosphate (FPP) synthase, thereby preventing the post-translational prenylation of small GTPases that are necessary for osteoclast polarisation, bone resorption, and cell survival [3,4]. Both simple BPs and nitrogen-containing BPs are therefore capable of causing osteoclast apoptosis, *in vitro *and *in vivo *[5], but by different molecular mechanisms. The regulation of osteoclast apoptosis appears to be an important mechanism of physiological bone homeostasis since a variety of growth factors and cytokines that stimulate bone resorption (such as receptor activator of nuclear factor-kappa B ligand [RANKL], interleukin-1, and tumour necrosis factor-alpha [TNF-α]) also prevent osteoclast apoptosis (reviewed elsewhere [6,7]). In this study, we examined the extent to which RANKL might antagonise the anti-resorptive activity of clodronate and alendronate *in vitro*. This is of particular relevance in the context of rheumatoid arthritis (RA), in which high levels of RANKL expressed by synovial fibroblasts and T lymphocytes contribute to osteoclast-mediated joint destruction [8-10]. Some BPs have been shown to prevent local and systemic bone loss in some animal models of inflammation-induced arthritis [11-14] and to preserve joint architecture in a recent clinical trial [15]. However, the effectiveness of BPs at preventing joint destruction in other clinical studies in patients with RA has been disappointing [16-19]. The reasons for this are not completely clear but could involve factors in the local environment of the inflamed joint, such as RANKL, that might antagonise the anti-resorptive action of BPs.

## Materials and methods

### Reagents

Clodronate (dichloromethylene-1,1-bisphosphonate) (CLO) and alendronate (4-amino-1-hydroxy-butylidene-1,1-bisphosphonate) (ALN) were kindly provided by Procter & Gamble Pharmaceuticals (Cincinnati, OH, USA). Stock solutions were prepared in phosphate-buffered saline (PBS) (the pH adjusted to pH 7.4 with 5 M sodium hydroxide) and filter-sterilised prior to use. Cell culture reagents were from Sigma-Aldrich (Poole, UK).

### Quantification of osteoclast apoptosis

Mature osteoclasts were isolated from rabbit long bones and seeded into 24-well plates as previously described [3]. The following day, the plates were washed several times with PBS to remove the majority of stromal cells, leaving cultures of approximately 95% pure osteoclasts (tartrate-resistant acid phosphatase [TRAP]-positive multinucleated cells). Cultures were incubated with alpha-minimum essential medium (α-MEM) containing 10% (vol/vol) fetal calf serum (FCS), 100 U/mL penicillin, 100 μg/mL streptomycin, and 100 μM CLO or ALN in the absence or presence of 100 ng/mL recombinant human RANKL (PeproTech, Rocky Hill, NJ, USA) (three wells per treatment). After 48 hours, the culture media were removed and adherent cells were fixed with 4% formaldehyde and either stained with 1 μg/mL 4,6-diamidino-2-phenylindole (DAPI) in PBS or stained for TRAP [20]. The number of TRAP-positive multinucleated osteoclasts per well or the proportion of osteoclasts with DAPI-stained nuclei showing characteristic apoptotic nuclear morphology (chromatin condensation and nuclear fragmentation) [21] was determined using a Zeiss Axiovert 135 microscope and × 20 objective (Carl Zeiss, Jena, Germany).

### Analysis of osteoclast morphology by scanning electron microscopy

Bone marrow cells from rabbit long bones were seeded onto discs of elephant ivory in 96-well plates [20] and cultured with α-MEM containing 10% (vol/vol) FCS with 50 μM CLO or ALN in the absence or presence of 100 ng/mL recombinant human RANKL. After 24 hours, cells were fixed in 2.5% (vol/vol) glutaraldehyde and 2.5 mM MgCl_2 _in 0.089 M phosphate buffer (pH 7.2) for 3 hours at room temperature. Discs were washed overnight in 0.1 M phosphate buffer (pH 7.2), post-fixed in osmium tetroxide for 1 hour, washed in distilled water, and dehydrated through a graded series of ethanol solutions. The samples were critical-point-dried from CO_2_, glued onto aluminium stubs with colloidal silver adhesive, sputter-coated with 20 nm platinum, and examined in a Jeol JSM-35CF scanning electron microscope (EM) (Jeol Ltd., Tokyo, Japan) operating at 10 kV.

### Quantification of osteoclast-mediated bone resorption

Rabbit bone marrow cells were seeded onto ivory discs as described above and cultured with α-MEM containing 10% (vol/vol) FCS with 100 μM CLO or ALN in the absence or presence of 100 ng/mL recombinant human RANKL (four wells per treatment). After 48 hours, the media were removed, cells were wiped from the ivory discs, and the total area of mineral resorbed per disc was determined using a reflected light microscope [3].

### Measurement of caspase-9 activity in osteoclasts

Rabbit osteoclasts, purified as described above, were cultured with α-MEM containing 100 μM ALN ± 100 ng/mL RANKL for 48 hours. Unfixed, adherent cells were stained using an Apofluor Green Caspase Activity Assay kit (Enzyme Systems Products, Livermore, CA, USA). This involves the covalent binding of a fluorescently labelled, cell-permeable caspase inhibitor to active caspase-9, thus allowing the detection of cells with caspase-9 activity. Cells were counterstained with Hoechst 33342, washed to remove excess stain, and visualised using a Zeiss Axiovert 135 microscope and × 20 objective.

### Western blot analysis

Mature osteoclasts were isolated from rabbit long bones, seeded into 10-cm-diameter Petri dishes, and purified as previously described [3]. Purified osteoclasts were cultured for 48 hours with 100 ng/mL RANKL or with 100 μM ALN ± 100 ng/mL RANKL (four dishes per treatment). Dishes were rinsed with PBS, and osteoclasts were lysed in 300 μL of RIPA buffer (1% [vol/vol] NP-40, 0.5% [wt/vol] sodium deoxycholate, and 0.1% [wt/vol] SDS) containing 20 μL of Sigma-Aldrich protease inhibitor cocktail (P-8340). Protein (40 μg) from each sample was electrophoresed under reducing conditions on a 12.5% polyacrylamide/SDS gel and then transferred onto polyvinyldifluoride membrane. Blots were hybridised with goat polyclonal anti-Rap1A (sc1482; Santa Cruz Biotechnology, Inc., Santa Cruz, CA, USA), rabbit polyclonal anti-Mcl-1 or mouse monoclonal anti-Bcl-2 (Santa Cruz Biotechnology, Inc.), anti-β-actin (Sigma-Aldrich), or rabbit polyclonal antibodies to cIAP-1, XIAP, or cIAP-2 (R&D Systems, Inc., Minneapolis, MN, USA), followed by horseradish peroxidise-conjugated secondary antibodies. Blots were visualised after chemiluminescence detection using a Bio-Rad FluorS Max imager (Bio-Rad Laboratories, Inc., Hercules, CA, USA).

### Generation and cytokine starvation of mouse osteoclasts

Mouse osteoclasts were generated *in vitro *from macrophage colony-stimulating factor (M-CSF)-dependent bone marrow macrophages. Bone marrow cells were flushed into 10-cm Petri dishes (Falcon, now part of BD Biosciences, San Jose, CA, USA) from the tibiae and femorae of adult male C57BL/6 mice and cultured in α-MEM containing 100 U/mL penicillin, 100 μg/mL streptomycin, 1 mM glutamine, 10% FCS, and 100 ng/mL murine M-CSF (R&D Systems, Inc.). After 2 days, non-adherent cells were removed and the adherent cells were re-seeded into 24-well plates (Corning Life Sciences, Acton, MA, USA) at a density of 2.5 × 10^4 ^cells per well in the medium described above containing 25 ng/mL M-CSF and 20 ng/mL murine RANKL (R&D Systems, Inc.). Multinucleated, TRAP-positive osteoclasts formed after 5 days.

These cultures are amenable to studies on osteoclast survival since (unlike rabbit osteoclasts) the cells are highly dependent on the presence of exogenous pro-survival factors such as M-CSF, RANKL, TNF-α, or lipopolysaccharide (LPS). To induce osteoclast apoptosis, the medium was removed and replaced with fresh medium lacking M-CSF/RANKL or with medium containing 100 ng/mL M-CSF, 100 ng/mL RANKL, 10 ng/mL TNF-α, 0.1 μM LPS, or M-CSF + RANKL (control). After 2 to 12 hours, cell lysates were analysed by Western blotting as described above using antibodies to Mcl-1, Bcl-2, Bcl-x_L _(Santa Cruz Biotechnology, Inc.), and cleaved caspase-3 (Promega Corporation, Madison, WI, USA). After 8 hours of cytokine starvation, osteoclasts grown on glass coverslips were also fixed and processed for analysis by scanning EM as described above.

### Statistical analysis

The effects of BPs and RANKL on osteoclast number, osteoclast apoptosis, bone resorption, and caspase-9 activity were analysed by analysis of variance with a Bonferroni *post hoc *test (SPSS version 9.0; SPSS Inc., Chicago, IL, USA).

## Results

### RANKL attenuates osteoclast apoptosis induced by clodronate or alendronate

**Figure 1:**
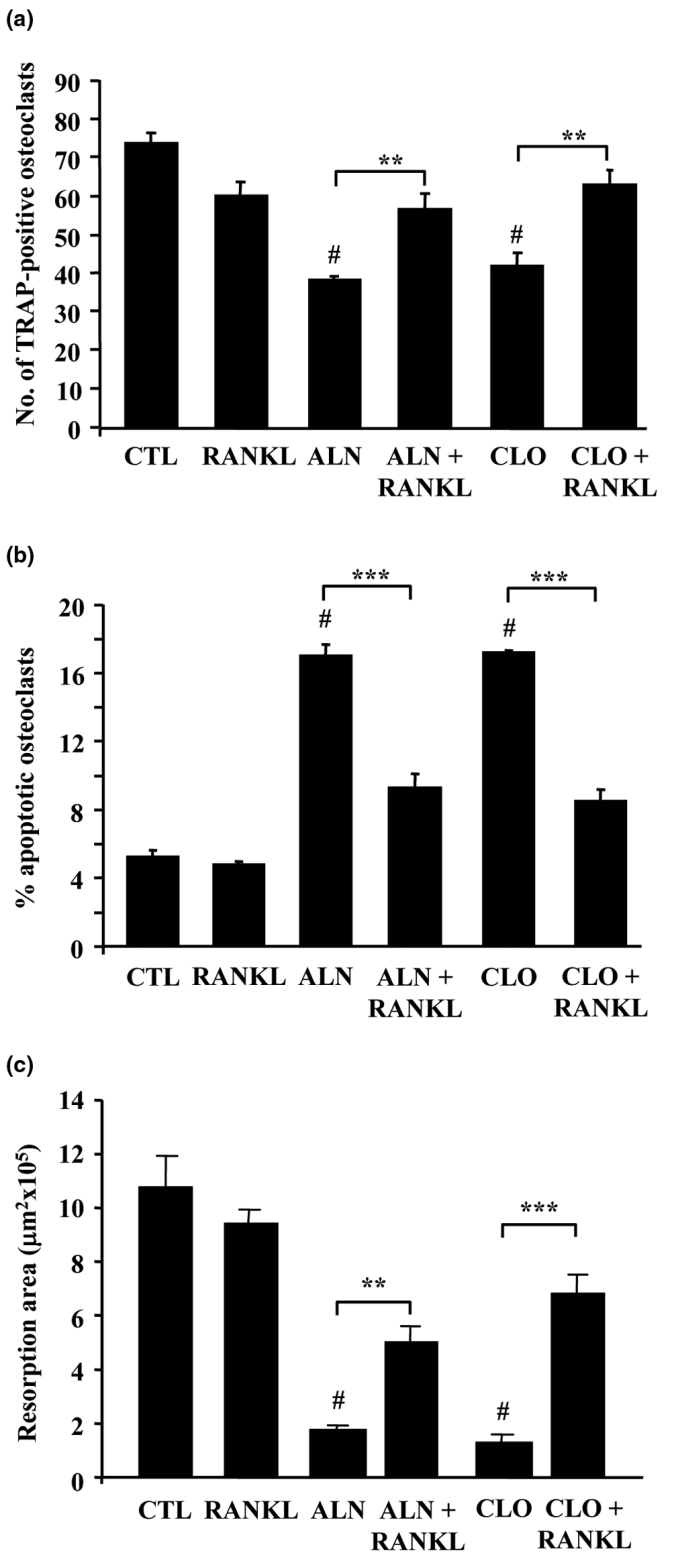
RANKL attenuates the effect of bisphosphonates on osteoclast number, apoptosis, and bone resorption *in vitro*. Cultures of mature osteoclasts from rabbit bones were treated with 100 μM ALN or CLO, ± 50 ng/mL RANKL for 48 hours. Cells were then fixed, stained for tartrate-resistant acid phosphatase (TRAP), and counterstained with DAPI. **(a) **Results are the mean number of TRAP-positive multinucleated osteoclasts (more than three nuclei per cell) ± standard error of the mean (SEM) (n = 3) or **(b) **the percentage of non-apoptotic and apoptotic osteoclasts. Data are expressed as the mean ± SEM (n = 3 replicates). **P = 0.01, ***P = 0.001 compared with ALN or CLO alone (analysis of variance). ^#^Treatment with ALN or CLO alone caused a significant decrease in osteoclast number compared with control (CTL) cultures (*P *= 0.01) and a significant increase in osteoclast apoptosis compared with control cultures (*P *= 0.001). **(c) **Values of resorption area are the mean resorbed area (mm^2^) per slice ± SEM (n = 6 slices). ***P = 0.001 compared with CLO alone and **P = 0.01 compared with ALN alone (analysis of variance). ^#^Treatment with ALN or CLO alone caused a significant decrease in osteoclastic bone resorption compared with control cultures (*P *= 0.001). The data shown are representative of three independent experiments. ALN, 4-amino-1-hydroxy-butylidene-1,1-bisphosphonate (alendronate); CLO, dichloromethylene-1,1-bisphosphonate (clodronate); DAPI, 4,6-diamidino-2-phenylindole; RANKL, receptor activator of nuclear factor-kappa-B ligand.

Treatment with 100 μM ALN or CLO reduced the number of adherent osteoclasts (TRAP-positive cells with at least three nuclei) in plastic culture dishes to approximately 52% and 57% of control cultures, respectively (Figure 1a). In the presence of 50 ng/mL RANKL, the reduction in osteoclast number was significantly attenuated (to approximately 77% and 85% of control cultures, respectively) (*P *< 0.01).

Osteoclasts in culture dishes that were undergoing apoptosis but remained adherent were identified on the basis of characteristic morphological features (chromatin condensation and nuclear fragmentation) after staining with DAPI [21]. Approximately 17% of adherent osteoclasts in culture dishes were apoptotic after treatment with 100 μM ALN or CLO for 48 hours. This was significantly reduced (to approximately 9%) in the presence of 50 ng/mL RANKL (*P *< 0.001), similar to the proportion of osteoclasts undergoing apoptosis in cultures without BPs (Figure 1b). RANKL alone did not significantly alter the number of adherent osteoclasts or the proportion of apoptotic osteoclasts in cultures in the absence of BPs.

### RANKL protects osteoclasts from the anti-resorptive effects of bisphosphonates

**Figure 2:**
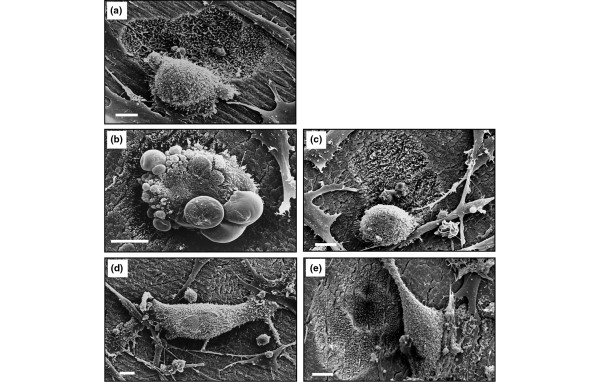
Scanning EM analysis of the effect of CLO, ALN, and RANKL on the morphology of mature osteoclasts cultured *in vitro*. Rabbit osteoclasts were cultured on ivory discs for 24 hours with 50 μM CLO or 50 μM ALN, ± 100 ng/mL RANKL. **(a) **Control. **(b) **CLO. **(c) **CLO + RANKL. **(d) **ALN. **(e) **ALN + RANKL. Osteoclasts were fixed and processed for scanning EM analysis. Bars = 10 μm. ALN, 4-amino-1-hydroxy-butylidene-1,1-bisphosphonate (alendronate); CLO, dichloromethylene-1,1-bisphosphonate (clodronate); RANKL, receptor activator of nuclear factor-kappa-B ligand.

The effect of RANKL on the morphology of BP-treated rabbit osteoclasts was also studied by scanning EM. After 24 hours of culture on ivory discs, 71% of osteoclasts in control cultures were spread on the mineral surface, often located in or adjacent to extensive and deep resorption cavities (Figure 2a). However, after treatment with 50 μM CLO for 24 hours, few, shallow resorption pits were present and 50% of the osteoclasts were rounded and lacked areas of spreading. Many of these rounded osteoclasts lacked membrane ruffles or microvilli but contained numerous blebs, indicative of cells undergoing apoptosis (Figure 2b). The morphology of other cell types, such as stromal cells, in the culture did not appear to be affected by CLO. The appearance of apoptotic osteoclasts was prevented by the presence of RANKL since 30% of osteoclasts were rounded when cultured with CLO + RANKL, few of these had membrane blebbing, and 70% of the osteoclasts appeared similar to those in control cultures, associated with numerous resorption pits (Figure 2c).

Treatment with 50 μM ALN for 24 hours caused the appearance of osteoclasts that (although still spread and adherent to the mineral) appeared retracted, with long cell processes (Figure 2d), and were associated (if at all) with only minor resorption pits. These osteoclasts often lacked microvilli but exhibited ridges on the basolateral membrane, and few (<5%) apoptotic osteoclasts were observed with the obvious membrane blebbing observed in CLO-treated cultures. After culture with ALN + RANKL, osteoclasts were mostly well spread with membrane ruffles and surface microvilli, and resorption cavities were more evident (Figure 2e). Some osteoclasts also retained the presence of membrane ridges on the basolateral surface, but few (<5%) had the retracted morphology of ALN-treated cells or the blebbed morphology of CLO-treated cells. As expected, when the area of bone resorption was quantified, 100 μM ALN or CLO significantly inhibited the resorptive activity of rabbit osteoclasts cultured on ivory discs *in vitro*. However, the inhibitory effect of ALN or CLO on bone resorption was significantly overcome by the presence of 100 ng/mL RANKL (Figure 1c).

### RANKL reduces caspase-9 activity in osteoclasts

**Figure 3:**
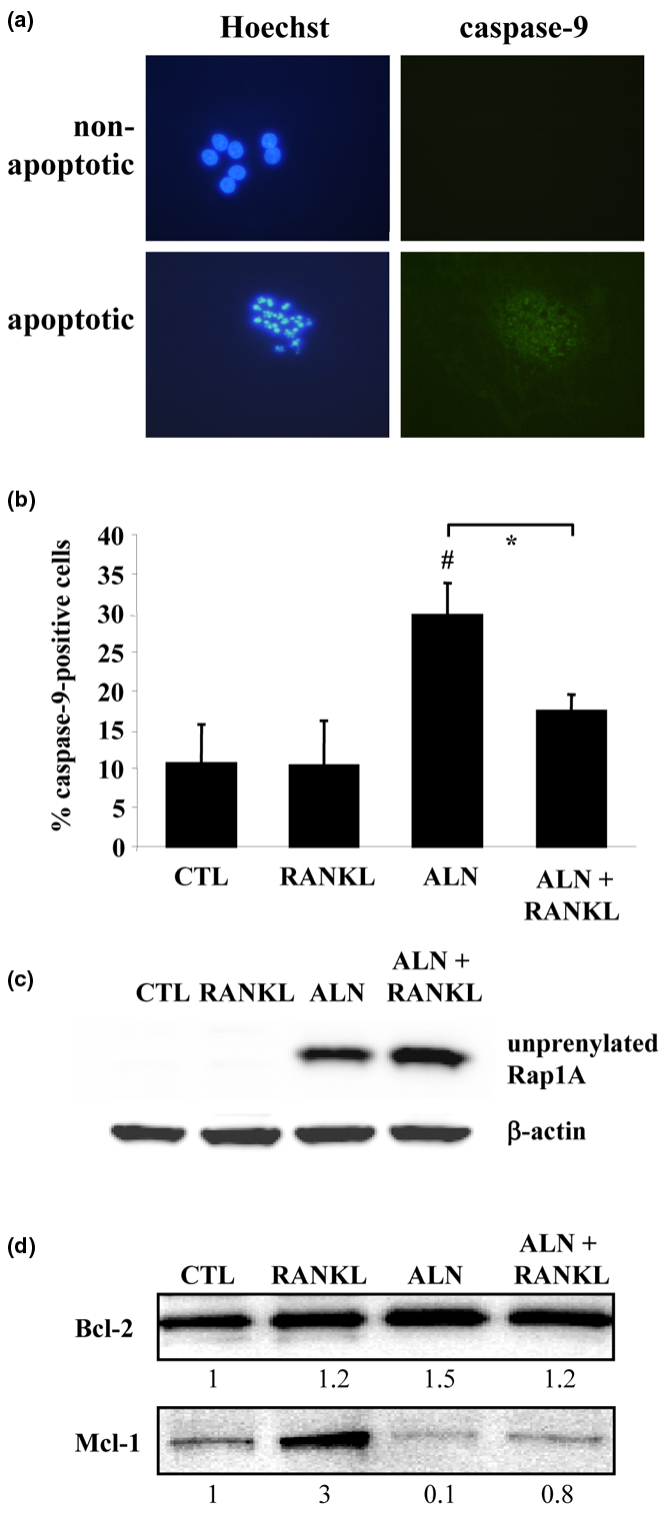
RANKL prevents activation of caspase-9 and increases Mcl-1 in osteoclasts but does not prevent inhibition of protein prenylation. Cultures of mature osteoclasts from rabbit bones were treated with 100 μM ALN ± 100 ng/mL RANKL for 48 hours and stained using an Apofluor Green Caspase-9 Activity Assay kit and Hoechst 33342. **(a) **A representative non-apoptotic and apoptotic osteoclast. **(b) **Quantification of caspase-9-positive osteoclasts after treatment with alendronate ± RANKL for 48 hours. *P ≤ 0.05 compared with ALN alone or ^#^*P *≤ 0.05 compared with control (CTL) (analysis of variance). Values are the mean ± standard error of the mean (n = 3 replicates) (100 to 150 cells counted per well). The data shown are representative of three independent experiments. **(c) **Purified rabbit osteoclasts were treated for 48 hours with 100 μM ALN ± 100 ng/mL RANKL or with RANKL alone. Cell lysates were then analysed by Western blotting for the unprenylated form of Rap1A and for β-actin. **(d) **Purified rabbit osteoclasts were treated for 48 hours with 100 μM ALN ± 100 ng/mL RANKL or with 100 ng/mL RANKL alone. Cell lysates were then analysed by Western blotting for Mcl-1 and Bcl-2. The level of Mcl-2 or Bcl-2 was quantified by densitometric analysis and expressed as a ratio of the level in control cells. Data shown are representative of three independent experiments. ALN, 4-amino-1-hydroxy-butylidene-1,1-bisphosphonate (alendronate); RANKL, receptor activator of nuclear factor-kappa-B ligand.

A fluorescent, cell-permeable caspase-9 inhibitor was used to identify single cells with caspase-9 activity (Figure 3a). In control cultures of purified rabbit osteoclasts, approximately 10% of the cells (Figure 3b) had detectable caspase-9 activity (similar to the proportion of cells in control cultures that were identified as apoptotic on the basis of nuclear morphology) (Figure 1b). After treatment with 100 μM ALN for 48 hours, the proportion of caspase-9-positive osteoclasts increased significantly (to about 30%). This was significantly reduced, almost to the proportion in control cultures, in the presence of 100 ng/mL RANKL.

### RANKL does not prevent accumulation of unprenylated Rap1A in osteoclasts

In accord with our previous studies, treatment of purified rabbit osteoclasts with 100 μM ALN for 48 hours caused the accumulation of the unprenylated form of the small GTPase Rap1A, thereby demonstrating that ALN inhibits protein prenylation in osteoclasts [2,22]. Incubation of osteoclasts with 100 μM ALN in the presence of 100 ng/mL RANKL for 48 hours did not prevent the accumulation of unprenylated Rap1A (Figure 3c).

### RANKL increases the level of Mcl-1 in rabbit osteoclasts

Western blot analysis of purified rabbit osteoclasts showed that treatment with 100 ng/mL RANKL, 100 μM ALN, or ALN + RANKL had no effect on the level of Bcl-2 protein (Figure 3d). However, RANKL alone consistently caused a threefold increase in the level of Mcl-1 protein in osteoclasts. Treatment with ALN caused a decrease of approximately 90% in Mcl-1, although co-treatment with RANKL almost completely prevented this effect and maintained the level of Mcl-1 similar to that in control cells (Figure 3d).

### Loss of Mcl-1 precedes apoptosis during cytokine deprivation of mouse osteoclasts but is prevented by pro-survival factors

**Figure 4:**
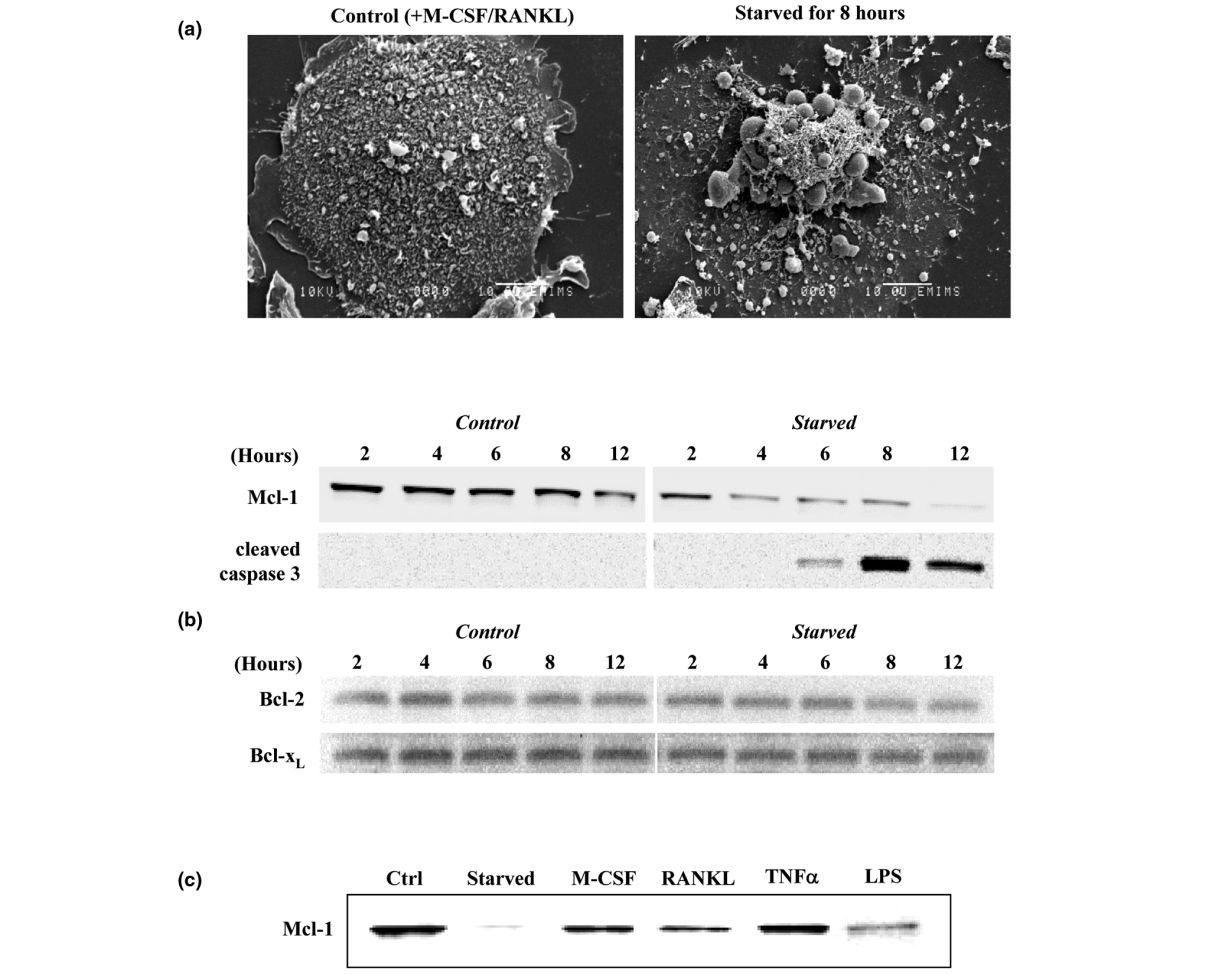
Mcl-1 levels decrease rapidly in mouse osteoclasts following cytokine starvation but are restored by pro-survival factors. Mouse osteoclasts were generated by culturing bone marrow macrophages for 5 days with macrophage colony-stimulating factor (M-CSF) + receptor activator of nuclear factor-kappa-B ligand (RANKL). **(a) **Osteoclasts were starved of M-CSF and RANKL for 8 hours and then fixed and processed for scanning EM analysis. Representative osteoclasts from non-starved (control) or starved cultures are shown. Bars = 10 μm. **(b) **Mouse osteoclasts were starved of M-CSF and RANKL for 2 to 12 hours. Western blot analysis was then used to determine the level of Mcl-1, cleaved caspase-3, Bcl-2, and Bcl-x_L _at each time point. **(c) **After osteoclasts were generated, the medium was replaced with normal medium (control [Ctrl]: M-CSF + RANKL), with medium lacking cytokines (starved), or with medium containing recombinant M-CSF, RANKL, tumour necrosis factor-alpha (TNF-α), or lipopolysaccharide (LPS). After 12 hours, Western blotting was used to determine the level of Mcl-1 in 40 μg of cell lysate. Data shown are representative of three independent experiments.

To further examine the importance of Mcl-1 in osteoclast survival, multinucleated osteoclasts were generated from M-CSF-dependent mouse bone marrow macrophages by culturing the latter cells for 5 days with M-CSF + RANKL. When the osteoclasts were starved of these cytokines, morphological changes indicative of apoptosis (Figure 4a) were apparent after 6 to 8 hours, consistent with the appearance in Western blots of the cleaved form of caspase-3 after 6 hours of cytokine starvation (Figure 4b). The appearance of apoptotic osteoclasts and cleaved caspase-3 was preceded by a decrease in the level of Mcl-1 (noticeable after 4 hours). Mcl-1 was almost completely absent after 12 hours of cytokine starvation, although the levels of Bcl-1 and Bcl-x_L _did not change during this time (Figure 4b). The loss of Mcl-1 that occurred in mouse osteoclasts following cytokine starvation could be prevented by the addition of M-CSF, RANKL, TNF-α, or LPS (Figure 4c).

## Discussion

BPs have become the mainstay of treatment for post-menopausal osteoporosis, Paget disease, and tumour-associated osteolysis and have been shown to prevent generalised bone loss in patients with RA treated with corticosteroids (reviewed recently by Breuil and Euller-Ziegler [16]). However, apart from a recent clinical study using the highly potent BP zoledronic acid in patients with RA [15] and two studies of zoledronic acid in animal models of RA [12,13], the effectiveness of BPs at preventing focal bone loss has been less convincing [16-19]. It has recently been suggested that the reason for this relative lack of effect on local, inflammatory bone loss is due to factors in the inflamed joint, such as TNF-α, that antagonise the ability of BPs to inhibit osteoclasts. Zhang and colleagues [23], using TNF-α transgenic mice, showed that Bcl-x_L _levels were markedly higher in osteoclasts, an effect that appeared to be caused by TNF-α-induced expression of Ets-2. Furthermore, overexpression of Ets-2 or Bcl-x_L _protected osteoclasts from ALN-induced apoptosis *in vitro*. RANKL is also abundant in the rheumatoid microenvironment and drives the enhanced osteoclastogenesis and hence excessive osteoclast-mediated destruction of bone [8-10]. In our study, we demonstrate that RANKL also protects osteoclasts from the apoptosis-inducing and anti-resorptive effects of ALN or CLO *in vitro*. The number of apoptotic rabbit osteoclasts was significantly lower in cultures treated for 48 hours with ALN or CLO in the presence of RANKL than in cultures treated with the BPs alone. Consistent with this, RANKL preserved the total number of osteoclasts in cultures treated with the BPs and also significantly overcame the anti-resorptive effect of the BPs when osteoclasts were cultured on dentine discs. This ability of RANKL to rescue osteoclasts from the effects of BPs was also observed morphologically by using scanning EM. Treatment of osteoclasts with CLO for 24 hours caused morphological changes associated with apoptotic cell death, consistent with the ability of CLO to induce osteoclast apoptosis via the formation and rapid accumulation of a cytotoxic metabolite [2,24,25]. The morphological changes associated with ALN treatment for 24 hours (cell retraction and alterations in plasma membrane morphology) are consistent with the inhibition of FPP synthase. This leads to the inhibition of protein prenylation and the slow accumulation of unprenylated small GTPases such as Rap1A [1,26]. The morphological appearance of osteoclasts after 24 hours of treatment with ALN therefore probably indicates cells with altered cytoskeletal arrangement and vesicular trafficking, as well as cells in the very early stages of apoptosis, as a result of abnormal small GTPase signalling [4,27]. In the presence of RANKL, these effects of ALN and CLO on osteoclast morphology were largely overcome, at least over a 24-hour culture period. In the case of ALN, this was not due to any ability of RANKL to prevent inhibition of FPP synthase, since RANKL treatment did not prevent the accumulation of unprenylated Rap1A in osteoclasts.

We have previously shown that BP-induced osteoclast apoptosis involves loss of mitochondrial membrane potential, leading (presumably as a result of subsequent release of cytochrome C from mitochondria and activation of procaspase-9) to the cleavage and activation of procaspase-3 [21]. To identify a potential mechanism by which RANKL prevents BP-induced apoptosis, we examined in more detail the events involved in ALN-induced apoptosis. Co-treatment with RANKL prevented the increase in the proportion of osteoclasts with active caspase-9 seen after ALN treatment, suggesting that RANKL either prevents the mitochondrial changes that lead to activation of procaspase-9 [28] or prevents procaspase-9 activation subsequent to the release of cytochrome C (for example, by increasing the expression of XIAP [29]). Although expression of XIAP and cIAP1/2 can be stimulated via nuclear factor-kappa-B [30], which is a major signalling pathway activated by RANKL [7], we did not observe any effect of RANKL on the level of XIAP, cIAP1, or cIAP2 in rabbit osteoclasts by Western blotting (data not shown), consistent with earlier studies on murine osteoclasts [31]. This suggests that RANKL probably prevents apoptosis by preventing mitochondrial changes prior to caspase activation, perhaps via members of the Bcl-2 family of proteins that regulate the mitochondrial pathway of apoptosis [32,33].

RANKL did not alter the level of Bcl-2 in rabbit osteoclasts but caused a marked increase in the level of Mcl-1, an anti-apoptotic member of the Bcl-2 family that is expressed in cells of the myeloid lineage such as macrophages and neutrophils [34,35], and is also known to be increased in synovial macrophages and fibroblasts in RA [36,37] as well as in synovial fluid lymphocytes in juvenile idiopathic arthritis [38]. In myeloid cells, Mcl-1 prevents apoptosis and its expression is highly regulated by survival-promoting factors via the PI3K/Akt/mammlian target of rapamycin (mTOR)/S6 kinase as well as mitogen-activated protein kinase (MAPK) signalling pathways [39,40]. The latter are also known to be activated by M-CSF, TNF-α, and RANKL [7,41], which promote osteoclast survival [6]. Whilst our work was in progress, Bradley and colleagues [42] showed that Mcl-1 mRNA and protein are upregulated in osteoclasts by M-CSF via activation of MEK/ERK (MAPK kinase/extracellular regulated kinase) and increased expression of Egr2. Hence, although the exact mechanism by which RANKL upregulates Mcl-1 in osteoclasts remains to be proven, it is highly likely that this also involves MAPK and/or mTOR signalling pathways. ALN treatment alone caused a decrease in Mcl-1 levels, perhaps since the activation of mTOR/S6 kinase in osteoclasts appears to involve geranylgeranylated proteins such as Rac [41], and ALN is known to effectively prevent protein geranylgeranylation [1,4]. With myeloma cells, others have also shown that the inhibition of protein geranylgeranylation causes the loss of Mcl-1 [43]. In our study, RANKL restored the level of Mcl-1 to that in control osteoclasts, but this was still less than the level of Mcl-1 in the presence of RANKL alone. Hence, ALN and RANKL may have opposing effects on the same signalling pathways (perhaps involving mTOR) that are required for Mcl-1 expression in osteoclasts.

In further support of an important role for Mcl-1 in osteoclast survival, we found that the level of Mcl-1 rapidly decreased following the removal of M-CSF and RANKL from cultures of mouse osteoclasts, which are highly dependent on the presence of such survival factors. In this model, the loss of Mcl-1 preceded the appearance of morphological and biochemical features of apoptosis and occurred in the absence of changes in the level of Bcl-2 or Bcl-x_L_. In addition, the loss of Mcl-1 in mouse osteoclasts could be prevented by the addition of factors (LPS, TNF-α, M-CSF, and RANKL) that are known to promote osteoclast survival [6,7,44]. The loss of Mcl-1 in osteoclasts probably occurred by proteasomal degradation since we also found that the proteasome inhibitor MG132 caused an increase in the level of Mcl-1 in mouse osteoclasts (data not shown). This is consistent with a requirement in osteoclasts for continual protein synthesis to prevent apoptosis [41,45]. Together, these observations indicate that Mcl-1 plays an important role in maintaining osteoclast survival.

Mcl-1 inhibits activation of the mitochondrial pathway of apoptosis by interacting with pro-apoptotic Bcl-2 family proteins such as Bak and Bim, thereby preventing the increased mitochondrial permeability that leads to caspase activation [33,46,47]. Lack of Bim leads to enhanced osteoclast survival and the pro-survival factor M-CSF decreases Bim levels in osteoclasts and osteoclast precursors [48,49], probably by increasing ubiquitin-dependent degradation of Bim via upregulation of cCbl [42]. The role of Bim in BP-induced osteoclast apoptosis is not known, although a recent study showed that apoptosis of MCF-7 breast cancer cells induced by the BP risedronate involved increased levels of Bim [50]. Upregulation of Mcl-1 (and therefore antagonism of Bim) by RANKL is therefore a likely mechanism by which RANKL prevents BP-induced apoptosis of osteoclasts.

## Conclusions

We have shown that RANKL protects osteoclasts from the pro-apoptotic and hence anti-resorptive effects of the BPs CLO and ALN *in vitro*. This protective effect of RANKL is likely to be mediated at least in part by an increase in the level of the anti-apoptotic protein Mcl-1. The ability of RANKL, together with other factors such as TNF-α [23], to rescue osteoclasts from the pro-apoptotic effects of BPs may account for the apparent lack of effectiveness of BPs (particularly the less potent BPs such as CLO and ALN) in preventing local, inflammation-induced bone loss in RA.

## Abbreviations

α-MEM: alpha-minimum essential medium; ALN: 4-amino-1-hydroxy-butylidene-1,1-bisphosphonate (alendronate); BP: bisphosphonate; CLO: dichloromethylene-1,1-bisphosphonate (clodronate); DAPI: 4,6-diamidino-2-phenylindole; EM: electron microscopy; FCS: fetal calf serum; FPP: farnesyl diphosphate; LPS: lipopolysaccharide; MAPK: mitogen-activated protein kinase; M-CSF: macrophage colony-stimulating factor; mTOR: mammalian target of rapamycin; PBS: phosphate-buffered saline; RA: rheumatoid arthritis; RANKL: receptor activator of nuclear factor-kappa-B ligand; TNF-α: tumour necrosis factor-alpha; TRAP: tartrate-resistant acid phosphatase.

## Competing interests

MJR has received research funding from Procter & Gamble (Cincinnati, OH, USA), Novartis (Basel, Switzerland), and Roche (Basel, Switzerland) and acts as a consultant for Novartis and Procter & Gamble. The other authors declare that they have no competing interests.

## Authors' contributions

MJR conceived and designed the study and wrote the manuscript. KAS, HLR, and DT helped to design, and performed, the experiments. All authors read and approved the final manuscript.
